# Inhibition of Cholera Toxin and Other AB Toxins by Polyphenolic Compounds

**DOI:** 10.1371/journal.pone.0166477

**Published:** 2016-11-09

**Authors:** Patrick Cherubin, Maria Camila Garcia, David Curtis, Christopher B. T. Britt, John W. Craft, Helen Burress, Chris Berndt, Srikar Reddy, Jessica Guyette, Tianyu Zheng, Qun Huo, Beatriz Quiñones, James M. Briggs, Ken Teter

**Affiliations:** 1 Burnett School of Biomedical Sciences, College of Medicine, University of Central Florida, Orlando, Florida, United States of America; 2 Department of Biology & Biochemistry, University of Houston, Houston, Texas, United States of America; 3 NanoScience Technology Center and Department of Chemistry, University of Central Florida, Orlando, Florida, United States of America; 4 Produce Safety and Microbiology Research Unit, Western Regional Research Center, United States Department of Agriculture - Agricultural Research Service, Albany, California, United States of America; New York State Department of Health, UNITED STATES

## Abstract

Cholera toxin (CT) is an AB-type protein toxin that contains a catalytic A1 subunit, an A2 linker, and a cell-binding B homopentamer. The CT holotoxin is released into the extracellular environment, but CTA1 attacks a target within the cytosol of a host cell. We recently reported that grape extract confers substantial resistance to CT. Here, we used a cell culture system to identify twelve individual phenolic compounds from grape extract that inhibit CT. Additional studies determined the mechanism of inhibition for a subset of the compounds: two inhibited CT binding to the cell surface and even stripped CT from the plasma membrane of a target cell; two inhibited the enzymatic activity of CTA1; and four blocked cytosolic toxin activity without directly affecting the enzymatic function of CTA1. Individual polyphenolic compounds from grape extract could also generate cellular resistance to diphtheria toxin, exotoxin A, and ricin. We have thus identified individual toxin inhibitors from grape extract and some of their mechanisms of inhibition against CT.

## Introduction

Several plant and bacterial toxins share a common structural organization that consists of a catalytic A subunit and a cell-binding B subunit. AB toxins include cholera toxin (CT) from *Vibrio cholerae*, Shiga toxin 1 (ST1) and Shiga toxin 2 (ST2) from *Escherichia coli* strains such as O157:H7, heat-labile toxin (LT) from enterotoxigenic *E*. *coli*, diphtheria toxin (DT) from *Corynebacterium diphtheriae*, exotoxin A (ETA) from *Pseudomonas aeruginosa*, and ricin from the plant *Ricinus communis*. These toxins are released into the extracellular milieu, but they act upon targets within the eukaryotic cytosol [1]. The toxins must therefore cross a membrane barrier in order to function. Some AB toxins, such as DT, access the cytosol from acidified endosomes [2,3]. Other AB toxins such as CT, ST1, and ST2 move from the plasma membrane to the endoplasmic reticulum (ER) before passage into the cytosol through a mechanism involving the quality control system of ER-associated degradation (ERAD) [4–6].

CT (S1 Fig) provides a well-characterized pathway for the intracellular trafficking and translocation of an AB toxin. The ring-like CTB homopentamer contacts GM1 gangliosides on the host plasma membrane, thereby triggering endocytosis through a lipid raft mechanism [7]. The internalized toxin then travels by retrograde vesicular transport from the endosomes, through the Golgi apparatus, and to the ER [8]. Reduction of the CTA1/CTA2 disulfide bond occurs in the ER and facilitates the subsequent chaperone-assisted separation of CTA1 from its holotoxin [9–12]. The dissociated CTA1 subunit shifts to a disordered state that activates its ERAD-directed export to the cytosol; interaction with several host factors in the cytosol allows CTA1 to regain an active, folded conformation that elevates intracellular cAMP through the ADP-ribosylation of Gsα [5,13].

Using a novel cell-based assay, we identified grape seed and grape pomace (skin) extracts as potent inhibitors of ST1 and ST2 [14]. We recently reported that grape extracts also block CT/LT intoxication of cultured cells and intestinal loops. The anti-CT properties of grape extract included (i) stripping pre-bound toxin from the cell surface; (ii) blocking the unfolding of the isolated A1 chain; (iii) disrupting the ER-to-cytosol export of CTA1; and (iv) inhibiting the catalytic activity of CTA1. Yet the extract did not affect toxin transport from the cell surface to the ER or the dissociation of CTA1 from its holotoxin [15]. A distinct subset of host-toxin interactions were thus disrupted by the application of grape extract, as opposed to a gross alteration of toxin or cellular function.

We hypothesized the polyphenolic constituents of grape extract [16–18] are a source of anti-toxin activity that function through the disruption of host-toxin interactions. To test this hypothesis, twenty individual phenolic compounds were screened for inhibitory effects against CT. We identified two compounds that prevent toxin binding to the plasma membrane, two that inhibit the enzymatic activity of CTA1, and four others that disrupt the cytosolic activity of CTA1 without directly affecting its enzymatic function. The two compounds that block toxin binding at the cell surface—epigallocatechin gallate (EGCG) and procyanidin B2 (PB2)—also strip pre-bound CT from the plasma membrane and are predicted to occupy the GM1 binding site of CTB by docking simulations. Additional toxicity assays with the purified polyphenols identified one compound that inhibited ricin, three that inhibited DT, and four that inhibited ETA. We have thus identified specific polyphenolic toxin inhibitors from grape extract and some of their mechanisms of action against CT.

## Materials and Methods

### Materials

Phenolic compounds were purchased in purified form from ChromaDex, Inc. (Irvine, CA). Individual polyphenols were solubilized in ethanol (quercitrin), methanol (kaempferol), methanol with 0.1% HCl (malvin and oenin), or water. Stock solutions were 2.5 mg/mL for all compounds other than cyanidin and quercitrin (1 mg/mL). Grape seed extract (stock solution of 10 mg/mL in water) was either purchased from ChromaDex or supplied by Polyphenolics, Inc. (Madera, CA). *E*. *coli* strain RM1697 was used for the production of a cell-free culture supernatant that contained both ST1 and ST2 [19]. Diethylamino(benzylidine-amino)guanidine (DEA-BAG) and protein disulfide isomerase were produced in the lab as previously described [11,15]. The purified CTA1/CTA2 heterodimer and a CTB pentamer conjugated with fluorescein isothiocyanate (FITC-CTB) were purchased from Sigma-Aldrich (St. Louis, MO). Ricin was purchased from Vector Laboratories (Burlingame, CA), while ETA, DT, and CT were purchased from List Biologicals (Campbell, CA). ST1 and a rabbit antibody against the A subunit of ST1 were obtained from BEI Resources (Manassas, VA).

### CT toxicity assay

CHO-K1 cells (ATCC #CCL-61) were co-incubated with a combination of CT and grape compound for 18 h before cAMP levels were quantified as previously described [15]. Unintoxicated cells were used to establish the basal levels of background cAMP, which were subtracted from each experimental value. Background-subtracted values were expressed as percentages of the maximum response from intoxicated but otherwise untreated CHO cells. All conditions were assessed with triplicate samples.

### Aggregation assay

Aggregated proteins exhibit larger hydrodynamic diameters than monomeric proteins. Dynamic light scattering was accordingly used to determine the hydrodynamic diameter of CT in the absence or presence of a phenolic compound. Toxin samples (1 mg/mL) were exposed to 10 μg/mL of the compound for 5 min at room temperature, and the samples were then added in 50 μL volumes to Sarstedt (Newton, NC) UV-transparent disposable cuvettes for measurement using a Zetasizer Nano ZS90 dynamic light scattering system (Malvern Instruments Ltd., England) equipped with a green (532 nm, 4 mW) laser and an Avalanche photodiode detector (quantum efficiency > 50% at 532 nm). The actual laser power used for the analysis of different sample solutions was adjusted by changing the attenuation numbers to obtain an optimum count rate around 100s kcps (kilo counts per seconds). Each sample was measured at least three times.

### Toxin cell binding assays

Vero cells (ATCC # CCL-81) grown overnight to ~80% confluency in clear-bottom, black-walled 96-well plates were incubated with 100 μL of FITC-CTB (1 μg/mL) or ST1 (0.5 μg/mL) in serum-free Ham's F-12 medium at 4°C for 30–60 min in the absence or presence of phenolic compound(s). Cells exposed to ST1 were washed with PBS, incubated with a rabbit antibody against the ST1 A subunit (1:500 dilution) for 60 min at 4°C, washed with PBS, and incubated with an AlexaFluor 488 donkey anti-rabbit IgG antibody (1:500 dilution) for 30 min at 4°C. All cells were washed extensively with PBS before a Synergy 2 plate reader was used to measure the fluorescence intensity using 485/20 nm excitation and 528/20 nm emission wavelength filters. A set of Vero cells incubated without toxin was used to establish the background level of autofluorescence that was subtracted from each experimental value. Background-subtracted values were expressed as percentages of the maximum signal obtained from cells incubated with the corresponding toxin in the absence of phenolic compound. At least six replicate wells were used for each condition.

### Computational studies

The study molecules were screened against the crystal structure of CT (PDB 1XTC) using Autodock Vina 1.1.2 with (x,y,z) center coordinates and sizes of (24,0,20.8) and (82,74,68), respectively. An exhaustiveness of 25 was used with a mode number of 50. The top 20 results were generated for each molecule. Random seeds were automatically assigned by Vina during the first round of docking, which involved five trials for each ligand using a search box that spanned the CT target. A second round of docking evaluations was completed using a focused box encompassing the putative binding sites identified from the first round at the base of the CT molecule, with a center of (2.0,0,22.8) and size of (46,74,68). Each trial was assigned an independent random seed. The 20 docking poses from each of the five trials were aggregated, and histograms generated for prevalent clusters (*n* = 100) in each of the four analysis systems.

### Transfected CTA1 translocation assay

CHO cells transfected with the pcDNA3.1/ssCTA1 plasmid were used to monitor CTA1 translocation from the ER to the cytosol as previously described [20]. With this system, an N-terminal signal sequence targets CTA1 for co-translational insertion into the ER lumen and is proteolytically removed from the mature CTA1 polypeptide after delivery to the ER. ER pellet fractions isolated from digitonin-permeabilized cells therefore contain two CTA1-specific bands: a slower migrating form corresponding to CTA1 with an intact signal sequence, and a faster migrating form representing the mature CTA1 polypeptide after cleavage of the signal sequence. Both forms were included in the quantification of ER-localized CTA1. Export efficiency was calculated by dividing the intensity of the CTA1 supernatant (i.e., cytosolic) band by the sum intensities of the pellet and supernatant bands. When indicated, 0.1 μM GA or 100 μg/mL of a phenolic cocktail was present during the methionine starvation and radiolabeling steps.

### Vero-d2EGFP toxicity assay

Ricin, ETA, DT, and ST1/ST2 intoxication assays were peformed with Vero-d2EGFP cells as previously described [21]. A set of unintoxicated parental Vero cells were used to establish the background level of autofluorescence, which was subtracted from each experimental value. The maximum fluorescent signal from Vero-d2EGFP cells incubated in the absence of toxin but with the corresponding grape compound (when appropriate) was set as the 100% value, and all corresponding values from toxin-treated cells were expressed as percentages of this value. In every experiment, at least six replicate wells were used for each condition.

## Results

### Identification of Specific Phenolic Compounds that Inhibit CT

In a previous study, we found a cocktail of 18 purified phenolic compounds conferred substantial cellular resistance to CT [15]. Each individual compound from that cocktail, as well as procyanidin B1 (PB1) and PB2, was screened for inhibitory effects against CT. High concentrations of individual polyphenolic compounds (>25 μg/mL) can induce non-specific protein aggregation [22–27], so the phenolic compounds were used at a concentration of 10 μg/mL (15–65 μM, as listed in Table 1). Toxin activity in the absence or presence of a phenolic compound was determined from the host cAMP response after an overnight challenge with 10 ng/mL of CT. Twelve purified compounds reduced the cAMP response to less than 50% of the control value from cells intoxicated in the absence of phenolic compound (Table 1). An MTS cell viability assay demonstrated none of the 12 hit compounds were toxic to CHO cells (S2A Fig). Furthermore, none of the compounds induced non-specific protein aggregation: boiled CT was detected in an aggregated state by dynamic light scattering, but aggregation did not occur when CT was incubated with 10 μg/mL of an individual phenolic compound (S2B Fig). Petunidin was not used in this aggregation assay because of the limited availability of the compound. In addition, none of the hit compounds substantially inhibited the forskolin-induced accumulation of cAMP (S2C Fig). Forskolin is an agonist of adenylate cyclase [28], so the anti-CT properties of our compounds could not be attributed to an inhibition of adenylate cyclase.

**Table 1:** Compound-induced alterations to host-toxin interactions involving CT.

	% Control Signal				
Compound (μM)	CT toxicity	FITC-CTB binding	Transfected CTA1 activity	Protein synthesis	In vitro CTA1 activity
Catechin (34)	98 ± 5	--	--	--	--
Oenin chloride (19)	*86 ± 11*	--	--	--	--
Malvin chloride (15)	*74 ± 18*	--	--	--	--
Epicatechin gallate (23)	74 ± 14	--	--	--	--
Peonidin (20)	*73 ± 3*	--	--	--	--
Protocatechin (65)	*72 ± 4*	--	--	--	--
Epicatechin (34)	67 ± 16	--	--	--	--
Catechin gallate (23)	63 ± 9	--	--	--	--
Petunidin (20)	*44 ± 2*	94 ± 3	--	--	--
Quercitrin (22)	41 ± 8	80 ± 6	55 ± 3	114 ± 11	78 ± 9
Caftaric acid (32)	*39 ± 9*	83 ± 8	98 ± 8	75 ± 11	45 ± 8
Kuromanin (21)	35 ± 15	78 ± 7	90 ± 17	84 ± 9	92 ± 5
Kaempferol (35)	35 ± 11	78 ± 7	10 ± 6	53 ± 6	53 ± 4
Procyanidin B2 (17)	31 ± 9	58 ± 8	63 ± 7	94 ± 10	100 ± 1
Procyanidin B1 (17)	*30 ± 3*	79 ± 9	81 ± 6	75 ± 9	69 ± 3
Gallic Acid (59)	*24 ± 1*	77 ± 5	89 ± 5	82 ± 9	107 ± 5
Resveratrol (44)	22 ± 9	80 ± 11	70 ± 10	70 ± 5	108 ± 3
Delphinidin (30)	*15 ± 5*	69 ± 7	29 ± 10	93 ± 12	76 ± 12
Cyanidin (31)	*10 ± 10*	79 ± 6	47 ± 9	87 ± 8	113 ± 15
Epigallocatechin gallate (22)	7 ± 3	47 ± 12	11 ± 9	66 ± 8	101 ± 6

To monitor CT toxicity, the levels of toxin-induced cAMP were quantified. To monitor toxin binding at the cell surface, CHO cells were incubated at 4°C for 1 h with FITC-CTB and a plant compound. To monitor the activity of cytosolic CTA1, a plasmid-based system expressed CTA1 directly in the cytosol of tranfected CHO cells. cAMP levels were recorded after a 4 h post-transfection chase in the presence of a plant compound. To monitor protein synthesis, cells were incubated with [^35^S]methionine following a 4 h incubation with a plant compound. The TCA-precipitated cpm from newly synthesized proteins were quantified by scintillation counting. To monitor *in vitro* CTA1 activity, DEA-BAG was used as a substrate for an ADP-ribosylation assay. For each assay, results were expressed as percentages of the control signal obtained from cells incubated in the absence of plant compound. Italicized numbers denote the averages ± ranges of two independent experiments. All other values represent the averages ± standard errors of the means (SEMs) of at least three independent experiments.

### Inhibition of CT Interaction with the Host Plasma Membrane

Grape extracts inhibit CT binding to the surface of a target cell [15]. This provides a possible mechanism for the compound-induced disruption of CT activity against cultured cells. As such, a fluorophore-labeled CTB pentamer (FITC-CTB) was used to monitor toxin binding to the host plasma membrane in the absence or presence of individual phenolic compounds. A combination of 1 μg/mL of FITC-CTB and 10 μg/mL of phenolic compound was added to cultured Vero cells at 4°C, a temperature that allows toxin binding to the cell surface but prevents endocytosis of the surface-bound toxin. Fluorescence was measured after 1 h. All results were expressed as percentages of the maximal signal obtained from cells incubated with FITC-CTB in the absence of grape compound. Control experiments established the phenolic compounds did not contribute an autofluorescent signal to this assay. Two compounds that generated a partial but statistically significant inhibition of FITC-CTB binding to the cell surface (*p* < 0.01, Student's t test) were chosen for further study: EGCG and PB2 (Table 1).

**Fig 1:**
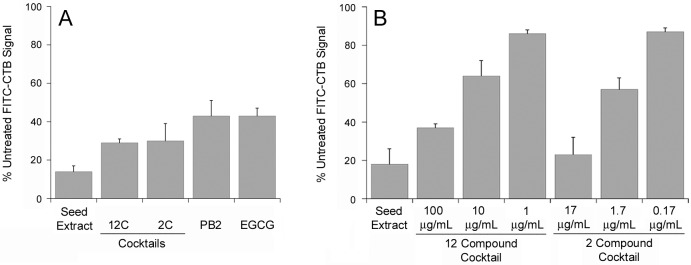
Polyphenolic compounds disrupt CT adherence to the host plasma membrane. (A) Vero cells were incubated at 4°C for 30 min with 1 μg/mL of FITC-CTB. Unbound toxin was removed from the medium and replaced with 100 μg/mL of grape seed extract, 100 μg/mL of a cocktail containing all 12 CT hit compounds (12C), 17 μg/mL of a cocktail containing PB2 and EGCG (2C), 10 μg/mL of PB2, or 10 μg/mL of EGCG. After an additional 30 min at 4°C, FITC-CTB fluorescence was recorded with a plate reader. Values were standardized to the FITC-CTB signal from control cells incubated in the absence of grape compounds. (B) Vero cells were incubated at 4°C for 1 h in the combined presence of FITC-CTB and 100 μg/mL of grape seed extract, various concentrations of the 12C cocktail, or various concentrations of the 2C cocktail. FITC-CTB fluorescence was then recorded, with values standardized to the FITC-CTB signal from control cells incubated in the absence of grape compounds. Data from both panels represent the means ± SEMs of 4 independent experiments with 6 replicate samples per condition.

Grape seed extract can also strip bound CT from the plasma membrane [15], so we examined whether EGCG and PB2 could remove FITC-CTB from the cell surface (Fig 1A). Vero cells incubated with 1 μg/mL of FITC-CTB for 30 min at 4°C were washed to remove unbound toxin and then exposed to grape compound for an additional 30 min at 4°C. After extensive washing, fluorescence from the surface-bound FITC-CTB was detected with a plate reader. This procedure was conducted with 10 μg/mL of EGCG (22 μM), 10 μg/mL of PB2 (17 μM), a 2 compound cocktail containing 8.5 μg/mL each of EGCG (19 μM) and PB2 (15 μM), a 12 compound cocktail containing all CT hit compounds at 8.5 μg/mL each, and, as a positive control, 100 μg/mL of grape seed extract. Cells incubated with FITC-CTB in the absence of grape compound were used as a control to establish the maximal FITC-CTB signal. EGCG and PB2 each reduced the FITC-CTB signal to about 40% of the control value, which was consistent with the results from our initial assay that involved co-application of FITC-CTB and grape compound to the cells (Table 1). The 2 and 12 compound cocktails could also strip pre-bound FITC-CTB from the plasma membrane, reducing the fluorescent signal to ~30% of the control value. Since the 12 compound cocktail was no more effective than the 2 compound cocktail, it appeared EGCG and PB2 were solely responsible for the disruption of toxin binding in the 12 compound cocktail and did not exhibit synergistic or antagonistic effects with the other phenolic compounds.

We next identified the effective concentrations of the 2 and 12 compound cocktails. For this experiment, cells placed at 4°C were co-incubated with FITC-CTB and grape compounds for 1 h before washing and measurement of fluorescent intensity. As shown in Fig 1B, both cocktails exhibited a dose-dependent inhibition of toxin binding to the cell surface. A 10-fold dilution of the highest cocktail concentration produced an approximately 2-fold reduction in the inhibitory effect, while a 100-fold dilution of the highest cocktail concentration minimized the inhibitory effect. A cocktail of EGCG and PB2 could therefore disrupt host-toxin interactions at a total polyphenol concentration of 1.7 μg/mL (0.85 μg/mL of each compound), but it was not effective at a lower concentration of 0.17 μg/mL.

The inhibition of CTB binding to the cell surface by EGCG and PB2 resulted from an interaction with the toxin rather than the host plasma membrane. This was demonstrated by incubating the cells with EGCG or PB2 for 30 min at 4°C. The cells were then washed to remove unbound compound and exposed to FITC-CTB for another 30 min at 4°C. With this protocol, neither EGCG nor PB2 substantially inhibited FITC-CTB binding to the plasma membrane (S3A Fig). It thus appeared that EGCG and PB2 were binding to the toxin rather than the host cell surface.

The specific interactions of EGCG and PB2 with CTB were further demonstrated with a ST1 binding assay. Vero cells were co-incubated with ST1 and 10 μg/mL of both EGCG and PB2 for 1 h at 4°C before toxin binding was assessed with a primary anti-ST A chain antibody and a FITC-conjugated secondary antibody. The fluorescent signal obtained from ST1 binding to EGCG- and PB2-treated cells was nearly equivalent to the signal obtained from its binding to untreated control cells (S3B Fig). Thus, in contrast to CT, EGCG and PB2 did not inhibit ST1 binding to the plasma membrane.

### Modeling of Compound Interaction with the CTB Pentamer

**Fig 2:**
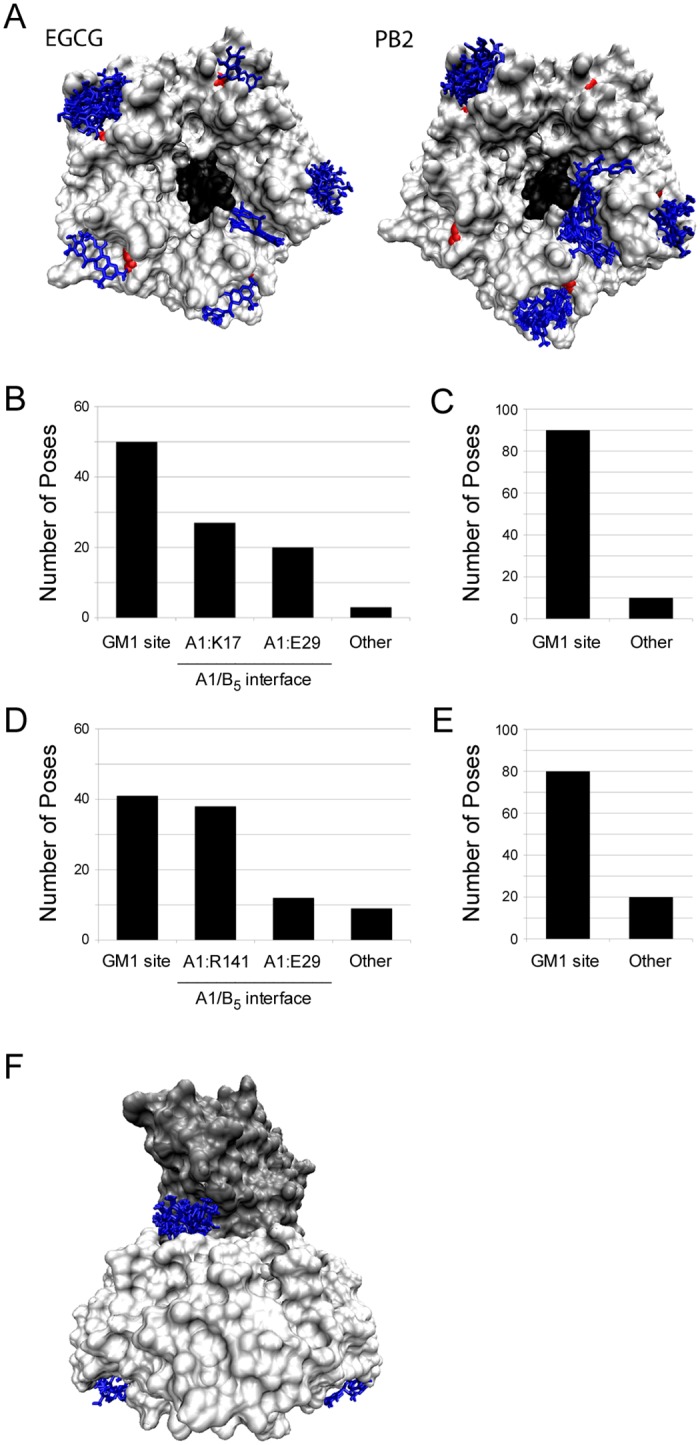
Docking simulations identify potential compound binding sites in CT and the CTB pentamer. Autodock Vina 1.1.2 was used to model the EGCG and PB2 binding sites on CT. (A) The top results for compound binding to CTB_5_ mapped to the base of the pentamer. Both images present a bottom-up view of CT. EGCG and PB2 are highlighted in blue; the CTA2 subunit is in black; and the CTB pentamer is white, with red indicating glycine 33 in the GM1 binding pocket of CTB. (B-C) The aggregate data from five trials of 20 poses each are presented for EGCG docking to (B) CT or (C) the CTB pentamer. (D-E) The aggregate data from five trials of 20 poses each are presented for PB2 docking to (D) CT or (E) the CTB pentamer. (F) PB2 docking to CT at the GM1 binding site and the CTA/CTB_5_ interface near CTA residue R141 is shown. PB2 is highlighted in blue; the CTB pentamer is in white, and CTA is in grey.

Consistent with our FITC-CTB studies, docking studies indicated EGCG and PB2 have favorable binding propensities for the host GM1 ganglioside binding pocket of CTB. Docked poses for the CT holotoxin (S4A and S4B Fig) clustered in the area of the GM1 binding site for both EGCG and PB2 (Fig 2A). In the aggregate of five trials, the largest cluster for EGCG included 50 poses around the GM1 binding site. Some poses also clustered in the A/B_5_ interface near CTA residues K17 and E29 (Fig 2B). When a secondary docking analysis was performed using a focused search space encompassing just the CTB pentamer (S4C and S4D Fig), the cluster around the GM1 binding site grew to 90 poses (Fig 2C). The clustering of poses for PB2 also showed a large group of 41 in the GM1 binding site (Fig 2D), with 80 members in the focused search space of the CTB pentamer (Fig 2E). PB2 also had a second substantial cluster of 38 poses in the A/B_5_ interface near CTA residue R141 (Fig 2F) and might therefore inhibit host-toxin interactions beyond CT binding to the plasma membrane. Combined with our cell-based assays, these computational studies strongly suggest EGCG and PB2 can inhibit CT activity against cultured cells by disrupting CTB interactions with its GM1 surface receptor.

### Compound Effects on CTA1 Translocation from the ER to the Cytosol

**Fig 3:**
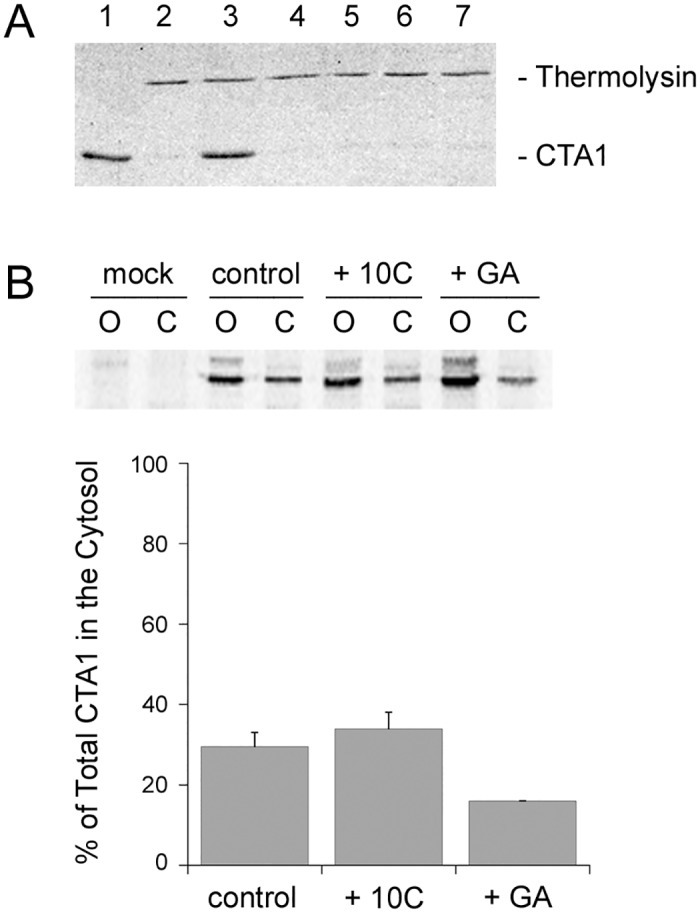
Phenolic compounds do not affect the thermal unfolding or ER-to-cytosol translocation of CTA1. (A) A purified CTA1/CTA2 heterodimer was placed in 20 mM sodium phosphate buffer (pH 7.4) containing 10 mM β-mercaptoethanol. Aliquots (1 μg) of the toxin were either left untreated (lanes 1–2), treated with 100 μg/mL of grape seed extract (lane 3), or treated with 10 μg/mL of a specific grape compound: caftaric acid (lane 4), quercitrin (lane 5), gallic acid (lane 6), or PB1 (lane 7). All samples were incubated at 37°C for 1 h. The samples were then shifted to 4°C and exposed to the protease thermolysin for 1 h, with the exception of the untreated toxin sample in lane 1. Samples were visualized by SDS-PAGE with Coomassie staining. Previous control experiments demonstrated that grape seed extract does not directly inhibit the proteolytic activity of thermolysin [15]. (B) Using a plasmid-based transfection system, CTA1 was co-translationally inserted into the ER lumen before export back into the cytosol. The intracellular distribution of CTA1 was determined by immunoprecipitation of organelle (O) and cytosol (C) fractions from transfected cells radiolabeled for 1 h in the absence of compound (control), in the presence of a phenolic cocktail (100 μg/mL) containing all CT hit compounds other than petunidin and resveratrol (10C), or in the presence of 0.1 μM GA. The percentage of radiolabeled CTA1 found in the cytosol was calculated from two independent experiments (averages ± ranges).

Grape extracts do not prevent retrograde CT transport from the plasma membrane to the ER or the ER-localized release of CTA1 from the rest of the toxin, but they do block the thermal unfolding and ER-to-cytosol export of CTA1 [15]. A protease sensitivity assay [15] was accordingly used to determine whether any of our hit compounds could stabilize CTA1 and thereby prevent its temperature-induced shift to an unfolded, protease-sensitive state. As shown in Fig 3A, the temperature-induced unfolding of CTA1 places the toxin in a protease-sensitive conformation (lane 2). Treatment with grape seed extract prevented the temperature-induced shift to a protease-sensitive conformation (lane 3), but no individual hit compound from the CT screen could replicate this effect (lanes 4–7, plus additional data not shown). A cocktail of all 12 compounds also failed to prevent the temperature-induced shift of CTA1 to a protease-sensitive conformation. Thus, none of our hit compounds (alone or in combination) held CTA1 in a folded conformation at physiological temperature. We also noted that none of the hit compounds inhibited reduction of the CT disulfide bond by protein disulfide isomerase (S5A Fig) and did not themselves reduce the CT disulfide bond (S5B Fig).

Thermal unfolding of the free, reduced CTA1 subunit places it in a translocation-competent conformation for ERAD-mediated export to the cytosol [5,13]. As our cocktail did not inhibit the temperature-induced shift of CTA1 to a protease-sensitive conformation, it would not block toxin translocation through a direct stabilizing effect on CTA1. However, the compounds could still inhibit toxin translocation through other mechanisms. To examine this possibility, we used a plasmid-based system to express CTA1 directly in the ER of transfected CHO cells. An N-terminal signal sequence targets this CTA1 construct for co-translational insertion into the ER, and the ER-localized toxin is then retro-translocated back into the cytosol [29,30]. To monitor CTA1 translocation, the toxin was immunoprecipitated from cytosolic and membrane fractions generated from transfected cells radiolabeled for 1 h in the absence or presence of a cocktail containing all anti-CT compounds (each at 10 μg/mL) other than petunidin and resveratrol. As shown in Fig 3B, 30% of radiolabeled CTA1 was found in the cytosolic fraction of untreated cells and 34% of radiolabeled CTA1 was found in the cytosolic fraction of cocktail-treated cells. Cells treated with the Hsp90 inhibitor geldanamycin (GA) contained 16% of total CTA1 in the cytosol, which represented an ~50% decrease from the untreated control condition. This GA-induced disruption of toxin translocation confirmed a previous observation [20] and served as a control for the inhibition of CTA1 export to the cytosol. Our collective observations thus demonstrated that neither the thermal unfolding nor the ER-to-cytosol export of CTA1 was inhibited by the cocktails of grape compounds.

### Compound-Induced Inhibition of CTA1 Enzymatic Activity

Compound-induced disruptions to CT intoxication could also result from an inhibitory effect on CTA1 activity. We examined this possibility with another plasmid-based CTA1 expression system, but in this case CTA1 was expressed directly in the cytosol of transfected cells. Previous studies have shown a cAMP response from CTA1-expressing cells can be detected as soon as 2 h post-transfection [30]. Compounds that disrupt toxin delivery to the cytosol would not affect this cAMP response because CTA1 is expressed directly in the cytosol and consequently bypasses the upstream trafficking and translocation events. Following a 3 h transfection, CHO cells were chased for 4 h in medium lacking or containing 10 μg/mL of a phenolic compound. Intracellular cAMP levels were then quantified. As shown in Table 1, three compounds (EGCG, kaempferol, and delphinidin) reduced the cAMP response of CTA1-expressing cells to ~30% or less of the control value from transfected cells chased in the absence of any compound. Another three compounds reduced the cAMP response to ~50% of the control value: PB2, quercitrin, and cyanidin. Multiple polyphenolic compounds thus appeared to interfere with the cytosolic activity of CTA1.

Protein synthesis is required for the plasmid-based expression of CTA1, so compounds that block protein synthesis could have an indirect inhibitory effect on the cAMP response to CTA1 expression. To control for this possibility, we followed an established protocol [14] to monitor the incorporation of [^35^S]methionine into newly synthesized proteins from cells incubated for 4 h in medium lacking or containing 10 μg/mL of a phenolic compound. CTA1 itself does not inhibit protein synthesis; its cytopathic effect is manifested through the ADP-ribosylation of Gsα and subsequent elevation of intracellular cAMP. As shown in Table 1, four of the six compounds that reduced the cAMP response from CTA1-expressing cells had minimal to no effect on protein synthesis: PB2, quercitrin, cyanidin, and delphinidin. The two compounds (EGCG and kaempferol) with greatest inhibitory activities against transfected CTA1 also had the greatest inhibitory effects on protein synthesis, yet the two effects were not proportional: protein synthesis was inhibited by 40–50%, whereas the cAMP response was inhibited by 90%. Other compounds such as resveratrol that had a comparable ~30% inhibitory effect on protein synthesis did not exhibit disproportional inhibitory effects on the cAMP response to CTA1 transfection. The minimal cAMP response from CTA1-expressing cells exposed to EGCG or kaempferol was therefore most likely due, at least in part, to an inhibitory effect on toxin activity rather than toxin synthesis.

To circumvent the problem of protein synthesis inhibition in our assessment of compound-induced effects on CTA1 enzymatic activity, we employed an alternative, *in vitro* assay to monitor CTA1 activity (Table 1). Samples of purified CTA1 were mixed with 10 μg/mL of a phenolic compound and DEA-BAG, a substrate for the ADP-ribosyltransferase activity of CTA1 [31]. Toxin activity from four replicate samples per condition was then monitored using the fluorescent signal resulting from substrate modification [15]. Two compounds reduced the *in vitro* enzymatic activity of CTA1 to ~50% of the control value: caftaric acid and kaempferol. Three other compounds (PB1, quercitrin, and delphinidin) had a weak inhibitory effect on *in vitro* CTA1 activity, and the remaining six compounds had no effect on the *in vitro* activity of CTA1.

The CT inhibitors could be further characterized based on their combined cellular and *in vitro* effects on CTA1 enzymatic activity (Table 1). Inhibitory effects from the *in vitro* ADP-ribosylation assay demonstrate a direct impact on CTA1 enzymatic activity, whereas inhibitory effects from the cell-based assay with transfected CTA1 could result from direct effects on CTA1 activity or indirect modulation of host-toxin interactions required for toxin activity in the cytosol. Four compounds (PB1, gallic acid, kuromanin, and resveratrol) exhibited weak to no inhibitory activity against CTA1 both in the cytosol and *in vitro*. Quercitrin exhibited roughly proportional cytosolic and *in vitro* inhibitory effects against CTA1. Two compounds (kaempferol and delphinidin) that strongly inhibited the cytosolic activity of transfected CTA1 had moderate inhibitory effects on CTA1 *in vitro*, and another three cellular inhibitors (EGCG, PB2, and cyanidin) did not block the *in vitro* activity of CTA1. One compound (caftaric acid) inhibited CTA1 activity *in vitro* but not in the cytosol. The discrepancies between our two assays for CTA1 activity likely reflect the more complex environment of the cell where host factors that modulate the cytosolic function of CTA1 [32–35] could either compensate for the compound-induced loss of *in vitro* activity or could be directly affected by the polyphenolic compounds. In the case of kaempferol, the combination of inhibiting *in vitro* toxin activity and host protein synthesis likely explains the dramatic disruption of transfected CTA1 activity. From these collective observations, it appears kaempferol and quercitrin directly inhibit CTA1 catalytic activity while EGCG, PB2, cyanidin, and delphinidin block the cytosolic activity of CTA1 without directly affecting the enzymatic function of CTA1.

### Several AB Toxins are Inhibited by Different Phenolic Compounds from Grape Extract

To determine if other AB toxins are also affected by grape seed extract, we challenged extract-treated cells with ricin, ETA, or DT. Because all three toxins inhibit protein synthesis, their activities were monitored through the loss of EGFP fluorescence in Vero-d2EGFP cells [21]. With this assay, we found cells were strongly protected against ricin (S6A Fig), ETA (S6B Fig), and DT (S6C Fig) when co-treated with the toxin and extract. Extract-treated cells were also highly resistant to ST1 and ST2 present in a cell-free *E*. *coli* culture supernatant (S6D Fig), which confirmed our previous observation [14] and demonstrated the anti-toxin property of grape seed extract was a reproducible effect that could be detected with distinct lots of extract. Control experiments ensured the grape extract itself did not contribute to the fluorescent signal.

Using the Vero-d2EGFP assay, each grape compound from our CT screen was assessed for inhibitory effects against ricin, ETA, DT, and ST1/ST2 (Table 2). We found EGCG provided statistically significant protection against an overnight challenge with 1 ng/mL of ricin, allowing cells to maintain 54% of the unintoxicated EGFP signal in comparison to 18% of the control signal generated by cells treated with ricin alone. EGCG was the only compound that conferred resistance to ricin, and it also conferred statistically significant protection against 100 ng/mL of ETA and 0.1 ng/mL of DT. Three other polyphenolic compounds also inhibited the cytotoxic activity of ETA (PB2, epicatechin gallate, and resveratrol), while two other compounds inhibited DT activity (PB1 and caftaric acid). No single compound inhibited the combination of ST1 and ST2, including 6 compounds at 10 μg/mL concentrations from a secondary Vero-d2EGFP screen: coumaric acid, ferulic acid, hydroxybenzoic acid, syringic acid, polydatin, and isorhamnetin (S7 Fig). EGCG has been reported to inhibit ST1 but not ST2 [26], which explains why it was not identified in our screen that challenged cells with a combination of ST1 and ST2. We found, however, that a cocktail of all 20 compounds from Table 2 (each at 5 μg/mL concentration) provided partial but statistically significant protection against ST1/ST2. This indicated additive effects from individual compounds could contribute to extract-induced toxin resistance.

**Table 2:** Inhibition of toxin activity by purified phenolic compounds.

		% Control EGFP Signal			
Compound	μM compound	Ricin	ETA	DT	ST1/ST2
No Treatment	--	18 ± 1	23 ± 3	18 ± 3	31 ± 2
Caftaric acid	32	25 ± 6	16 ± 3	**46 ± 11**	28 ± 1
Catechin	34	22 ± 5	22 ± 3	20 ± 3	33 ± 8
Catechin gallate	23	22 ± 5	15 ± 2	19 ± 2	39 ± 8
Cyanidin	31	14 ± 2	14 ± 3	13 ± 3	30 ± 8
Delphinidin	30	17 ± 2	15 ± 6	31 ± 8	34 ± 8
Epicatechin	34	20 ± 4	16 ± 2	18 ± 5	30 ± 5
Epicatechin gallate	23	17 ± 5	**73 ± 12**	28 ± 5	38 ± 2
Epigallocatechin gallate	22	**54 ± 10**	**85 ± 7**	**59 ± 3**	40 ± 4
Gallic acid	6	28 ± 6	15 ± 6	33 ± 7	27 ± 4
Kaempferol	35	15 ± 4	20 ± 7	19 ± 3	32 ± 5
Kuromanin	21	19 ± 5	9 ± 3	20 ± 1	24 ± 3
Malvin	15	17 ± 3	28 ± 8	14 ± 2	29 ± 6
Oenin	19	15 ± 2	29 ± 7	17 ± 1	27 ± 5
Peonidin	20	20 ± 3	27 ± 9	14 ± 1	30 ± 8
Petunidin	20	18 ± 3	13 ± 1	15 ± 1	31 ± 12
Procyanidin B1	2	14 ± 2	18 ± 3	**46 ± 9**	25 ± 4
Procyanidin B2	2	16 ± 3	**66 ± 11**	15 ± 5	27 ± 2
Protocatechin	65	12 ± 3	13 ± 4	8 ± 4	29 ± 7
Quercitrin	22	14 ± 1	18 ± 5	9 ± 4	22 ± 3
Resveratrol	4	20 ± 4	**42 ± 5**	10 ± 2	33 ± 8
20 compound cocktail	--	--	--	--	**52 ± 5**

Vero-d2EGFP cells incubated with the listed concentrations of phenolic compound were challenged overnight with 1 ng/mL of ricin, 100 ng/mL of ETA, 0.1 ng/mL of DT, or a 1:10 dilution of a ST1/ST2-containing cell-free culture supernatant from *E*. *coli* strain RM1697. The fluorescent signal from toxin-challenged cells was expressed as a percentage of the control EGFP signal recorded for unintoxicated cells incubated with the relevant phenolic compound. "No Treatment" refers to toxin-challenged cells incubated in the absence of phenolic compound. Data represent the means ± SEMs of at least 4 independent experiments with 6 replicate samples. Bold numbers indicate statistically significant differences from the relevant No Treatment value (*p* < 0.02, Student's t test).

Because EGCG alone was effective against four of the five tested toxins, we focused additional attention on EGCG and generated dose response curves for its inhibitory action against CT, ricin, ETA, and DT (S8 Fig). At the half-maximal effective dose of toxin, we found 1 μg/mL of EGCG (2.2 μM) provides 4-fold cellular resistance to DT; 7-fold resistance to ricin; 15-fold resistance to CT; and 50-fold resistance to ETA.

## Discussion

Each AB toxin exploits a distinct subset of surface receptors, intracellular trafficking/translocation mechanisms, and cytosolic targets [1]. It is therefore difficult to block the cellular effects of multiple AB toxins with a single inhibitory agent. Grape extract, however, has now been shown to inhibit at least six different AB toxins: ricin, ETA, DT, ST1/ST2, CT, and LT (S6 Fig) [14,15]. This broad-spectrum effect can be attributed, at least in part, to the heterogeneous mixture of polyphenolic compounds in the extract. For example, we identified caftaric acid as an inhibitor of DT and CT; epicatechin gallate as an inhibitor of ETA; and EGCG as an inhibitor of DT, CT, ETA, and ricin. When all these individual compounds are present in an extract or a defined cocktail of compounds, they would comprise a broad-spectrum toxin inhibitor.

In a recent report by the USDA/Agricultural Research Service, scientific findings were documented on the development of alternative and novel approaches that employ plant-derived compounds as effective antimicrobials in food production [36]. In particular, current research is investigating the use of phytochemicals, composed of a wide variety of bioactive polyphenolic and terpenoid compounds [37,38], as food additives to improve food safety and benefit food animal production. Our works suggests the polyphenolic constituents of grape extracts, which are generally recognized as safe and sold as nutritional supplements, could potentially be used for this purpose as a broad-spectrum inhibitor of enteric toxins.

Our toxicity assays demonstrated individual phenolic compounds can protect cultured cells from specific toxins or subsets of toxins. This observation alone suggested each identified inhibitor acted through a defined mechanism: a global, non-specific inhibitory effect would be expected to affect all tested toxins rather than an individual toxin or subset of toxins. In addition, epicatechin gallate—which differs from EGCG by a single hydroxyl group—only inhibited ETA whereas EGCG inhibited DT, CT, ETA, and ricin. The different impact of epicatechin gallate and EGCG on intoxication strongly suggests the specific structure of each polyphenol (as opposed to a general property of polyphenols) is responsible for its anti-toxin properties. Control experiments further ensured the grape compounds did not (i) contribute an autofluorescent signal to the Vero-d2EGFP assay; (ii) inhibit the production of cAMP resulting from forskolin treatment; (iii) induce toxin aggregation; or (iv) affect cell viability. These observations provided additional evidence for the specific, non-toxic mode of action for our slate of toxin inhibitors.

We elucidated some of the molecular mechanisms for compound-induced resistance to CT. Different compounds had different effects on host-CT interactions, which again suggested each CT inhibitor had a specific mode of action. No compound affected the thermal stability of CTA1, activity of the thermolysin protease, reduction of the CT disulfide bond by protein disulfide isomerase, or the ER-to-cytosol export of CTA1 which involves several host factors of the ERAD system. However, we found that EGCG and PB2 could, like other plant compounds [24,27,39–41], prevent toxin binding at the cell surface. We further demonstrated EGCG and PB2 could strip bound CTB from the host plasma membrane. Docking simulations suggested this effect results from disruption of the CTB interaction with its GM1 receptor. EGCG and PB2 could thus serve as starting points for structure-activity relationship studies to generate new inhibitory agents for the prevention and treatment of cholera. It should be noted that EGCG and PB2 did not induce CT aggregation at the 10 μg/mL (~20 μM) concentration used in our CT studies, did not directly interact with the host plasma membrane, and did not inhibit ST1 binding to the cell surface. EGCG and PB2 thus appear to specifically disrupt CT-GM1 interactions, in contrast to the inhibition of LT-GM1 interaction resulting from toxin precipitation with a minimum of 75 μg/mL (165 μM) EGCG [27].

Resveratrol, one of our 12 CT inhibitors, has been shown to block CT activity against Vero cells through disruptions of toxin internalization and toxin activity. Both effects were observed in the presence of 100 μg/mL (0.4 mM) resveratrol and could be attributed to the partial precipitation of CT by resveratrol [25]. In contrast, a 10-fold lower concentration of resveratrol did not induce CT aggregation/precipitation (data not shown) [25] and did not inhibit *in vitro* CTA1 catalytic activity (Table 1) [25]. These observations indicate the mode of toxin inhibition will depend upon the concentration of applied polyphenol, with high concentrations producing non-specific effects. Morinaga, Yahiro, and Noda [25] did not detect a protective anti-toxin effect using 50 μg/mL (0.2 mM) or less of resveratrol, whereas we recorded an 80% loss of toxicity with just 10 μg/mL (44 μM) of the compound (Table 1). This apparent discrepancy likely reflects the different intoxication conditions of the two studies (1.6 μg/mL of CT for 30 min vs. 10 ng/mL of CT for 18 h in our assay) and suggests the protective effect of 44 μM resveratrol can be overwhelmed by high toxin concentrations.

Two compounds (kaempferol and quercitrin) appeared to directly inhibit the catalytic activity of CTA1, which has been observed for other plant products as well [42,43]. Other polyphenolic compounds conferred resistance to CT by disrupting host-toxin interactions required for the cytosolic activity of CTA1. For each of these events, the exact molecular mechanism remains to be determined and could involve a number of cellular effects. For example, an inhibition of cytosolic CTA1 activity could involve alterations to CTA1 interactions with Hsp90, ADP-ribosylation factors, Gsα, or lipid rafts [32–35,44]. It is also possible that a single compound could affect multiple steps of the intoxication process, such as the inhibition of both CTB surface binding and CTA1 cytosolic activity by EGCG.

The inhibitory constituents of grape extract were not identified in our previous studies. Here, we have shown a subset of polyphenolic compounds present in grape extract inhibit CT and other AB-type protein toxins. Furthermore, a general mode of inhibitory action against CT was identified for six compounds. This information should facilitate studies on the cell biology of intoxication through the application of new reagents to alter defined events in the intoxication process. Golgicide A, for example, was isolated in a high-throughput screen for ST1 inhibitors and has been used to study toxin biology and the cell biology of vesicular transport [45]. The identification of specific toxin inhibitors from grape extract can also serve as the foundation for structure-activity relationship studies to understand the molecular details of specific host-toxin interactions. Finally, this work provides a possible foundation for the use of natural products in the formulation of a broad-spectrum, food-compatible toxin inhibitor.

## Supporting Information

S1 Fig CT structure. CT is an AB_5_-type protein toxin composed of a catalytic A1 subunit (green), an A2 linker (purple), and a cell-binding B homopentamer (blue). The A1 and A2 subunits are initially synthesized as a single CTA polypeptide that undergoes proteolytic nicking to generate separate A1 and A2 subunits which remain linked by a disulfide bond (yellow). Reduction of the CTA1/CTA2 disulfide bond and separation of CTA1 from CTA2/CTB_5_ precede CTA1 export to the cytosol where it elicits a cytopathic effect. PDB 1S5F.
(TIF)

S2 Fig Plant phenols do not affect cell viability, protein aggregation, or adenylate cyclase activity. (A) CHO cells were incubated for 18 h with 10 μg/mL of the indicated compound or 20% DMSO before cell viability was determined with an MTS assay. Results were expressed as percentages of the MTS signal from untreated CHO cells. Data represent the avgs. ± std. devs. of 3 experiments or avgs. ± ranges of 2 experiments for kaempferol, procyanidin B2, delphinidin, EGCG, and DMSO. (B) The hydrodynamic diameters of CT (red), CT mixed with 10 μg/mL EGCG (blue) or procyanidin B2 (black), or boiled CT (green) were assessed by dynamic light scattering. As shown for EGCG and procyanidin B2, none of the tested compounds altered the hydrodynamic size of CT. (C) CHO cells were incubated with forskolin and 10 μg/mL of the indicated compound for 2 h before detecting the adenylate cyclase-driven production of cAMP.
(TIF)

S3 Fig EGCG and PB2 do not inhibit CT through direct binding to the plasma membrane and do not inhibit ST1 binding to the plasma membrane. (A) Vero cells were incubated at 4°C for 30 min with 10 μg/mL of EGCG or PB2. The polyphenol was then removed from the medium and, after several washes, replaced with 1 μg/mL of FITC-CTB. After an additional 30 min at 4°C, unbound toxin was removed and FITC-CTB fluorescence was recorded with a plate reader. Values were standardized to the FITC-CTB signal from control cells that were not incubated with EGCG or PB2. (B) Vero cells were incubated for 1 h at 4°C with 0.5 μg/mL of ST1 and a cocktail containing 10 μg/mL each of EGCG and PB2. After subsequent incubations with anti-ST primary and AlexaFluor 488-conjugated secondary antibodies, the extent of ST1 binding was determined by fluorescent measurement with a plate reader. Values were standardized to the fluorescent signal from control cells that were exposed to ST1 in the absence of EGCG and PB2. Data from both panels represent the means ± SEMs of 3–4 independent experiments with 6 replicate wells per condition.
(TIF)

S4 Fig CT search boxes. (A-B) The CT docking search box was defined by an unbiased large box (red) with center coordinates and sizes of (24, 0, 20.8) and (82, 74, 68), respectively. Panel B is rotated 90 degrees in relation to panel A. (C-D) A second round of docking used a more focused search box (blue) defined with center of (2.0, 0, 22.8) and size of (46, 74, 68). Panel D is rotated 90 degrees relative to panel C.
(TIF)

S5 Fig Phenolic compounds do not affect reduction of the CT disulfide bond. (A) CT was incubated with protein disulfide isomerase (PDI) for 1 h at 25°C in the presence of individual phenolic compounds before non-reducing SDS-PAGE with Coomassie staining was used to assess the redox status of the CTA subunit. Reduction of the CTA disulfide bond generates a 21 kDa CTA1 subunit and a 5 kDa CTA2 subunit; the CTB monomer is 11.5 kDa. Lane 1, CT alone; lanes 2–12, CT + PDI without added polyphenol (lane 2) or with 10 μg/mL PB2 (lane 3), kuromanin (lane 4), kaempferol (lane 5), gallic acid (lane 6), resveratrol (lane 7), quercitrin (lane 8), delphinidin (lane 9), cyanidin (lane 10), EGCG (lane 11), or PB1 (lane 12). (B) CT was incubated in the presence of individual phenolic compounds (10 μg/mL) for 1 h at 25°C before non-reducing SDS-PAGE with Coomassie staining was used to assess the redox status of the CTA subunit. Lane 1, untreated CT; lanes 2–12 CT treated with PB2 (lane 2), kuromanin (lane 3), kaempferol (lane 4), gallic acid (lane 5), resveratrol (lane 6), quercitrin (lane 7), delphinidin (lane 8), cyanidin (lane 9), EGCG (lane 10), PB1 (lane 11), or, as a positive control, β-mercaptoethanol (lane 12).
(TIF)

S6 Fig Grape extract confers cellular resistance to multiple AB toxins. Vero-d2EGFP cells were co-incubated for 18 h in the absence (circles) or presence (squares) of 100 μg/mL of grape seed extract and various concentrations of (A) ricin, (B) ETA, (C) DT, or (D) ST1 and ST2 present in the cell-free culture supernatant of *E*. *coli* strain RM1697. For each experiment, results from six replicate wells per condition were expressed as percentages of the maximal EGFP signal recorded for unintoxicated Vero-d2EGFP cells. Data represent the means ± SEMs of at least 4 independent experiments for each toxin.
(TIF)

S7 Fig Secondary screen for ST1/ST2 inhibitors. Vero-d2EGFP cells incubated with the listed concentrations of phenolic compound were challenged overnight with a ST1/ST2-containing cell-free culture supernatant from *E*. *coli* strain RM1697. The fluorescent signal from toxin-challenged cells was expressed as a percentage of the control EGFP signal recorded for unintoxicated cells incubated with the relevant phenolic compound. "No treatment" refers to toxin-challenged cells incubated in the absence of phenolic compound. Data represent the avgs. ± std. devs. of 3 independent experiments with 6 replicate samples.
(TIF)

S8 Fig EGCG confers cellular resistance to multiple AB toxins. (A) CHO cells were exposed to varying concentrations of CT for 18 h in the absence or presence of EGCG before intracellular cAMP levels were quantified. Results from 3 replicate wells per condition were expressed as percentages of the maximal cAMP response from CHO cells incubated with 10 ng/mL of CT in the absence of EGCG. (B-D) Vero-d2EGFP cells incubated in the absence or presence of EGCG were challenged for 18 h with various concentrations of (B) ricin, (C) ETA, or (D) DT. Results from six replicate wells per condition were expressed as percentages of the maximal EGFP signal recorded for unintoxicated Vero-d2EGFP cells incubated with the corresponding concentration of EGCG. Circles, no EGCG present; squares, 4 μg/mL (8.8 μM) EGCG; inverted triangles, 1 μg/mL (2.2 μM) EGCG; diamonds, 0.5 μg/mL (1.1 μM) EGCG; triangles, 0.1 μg/mL (0.2 μM) EGCG. Data represent the means ± SEMs of at least 3 independent experiments for each toxin.
(TIF)
