# Supplementary figures and images for: Inhibition of Cholera Toxin and Other AB Toxins by Polyphenolic Compounds
Source: PLoS One. 2016 Nov 9;11(11):e0166477. doi: 10.1371/journal.pone.0166477 (PMC5102367; doi:10.1371/journal.pone.0166477)

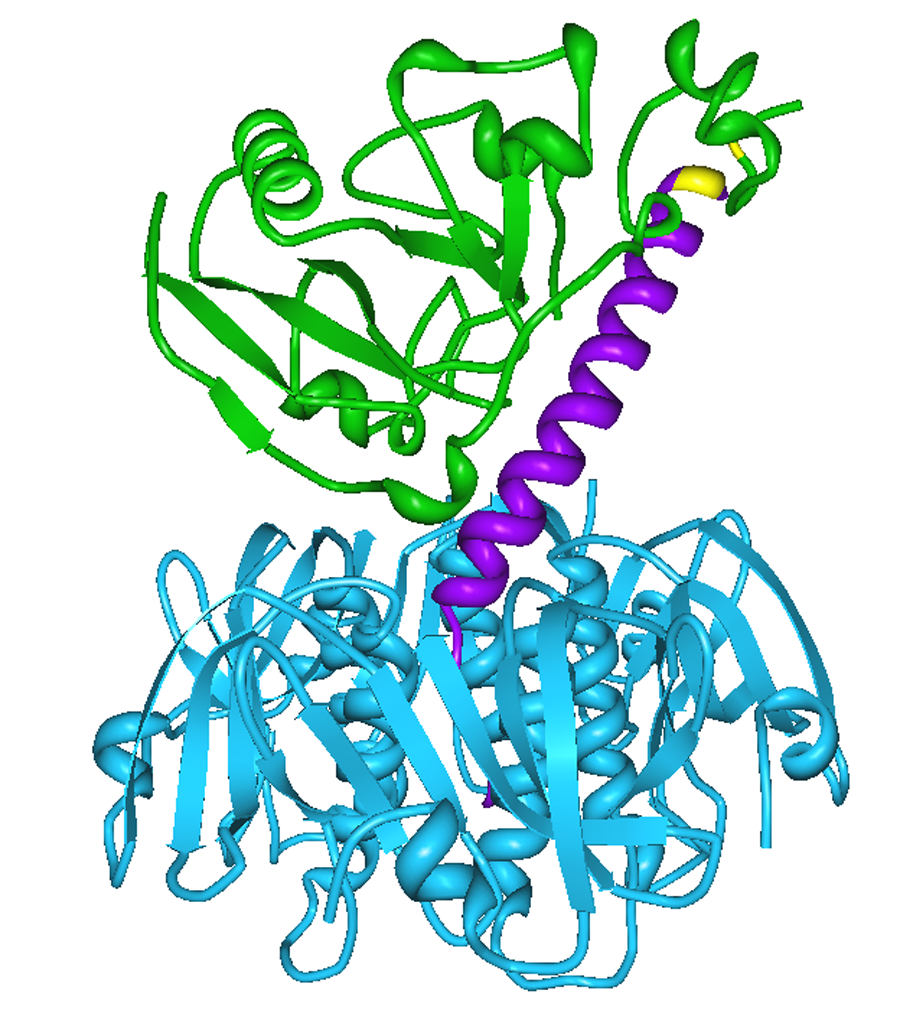

Supplement: S1 Fig — CT is an AB5-type protein toxin composed of a catalytic A1 subunit (green), an A2 linker (purple), and a cell-binding B homopentamer (blue). The A1 and A2 subunits are initially synthesized as a single CTA polypeptide that undergoes proteolytic nicking to generate separate A1 and A2 subunits which remain linked by a disulfide bond (yellow). Reduction of the CTA1/CTA2 disulfide bond and separation of CTA1 from CTA2/CTB5 precede CTA1 export to the cytosol where it elicits a cytopathic effect. PDB 1S5F. (TIF) [file pone.0166477.s001.tif]

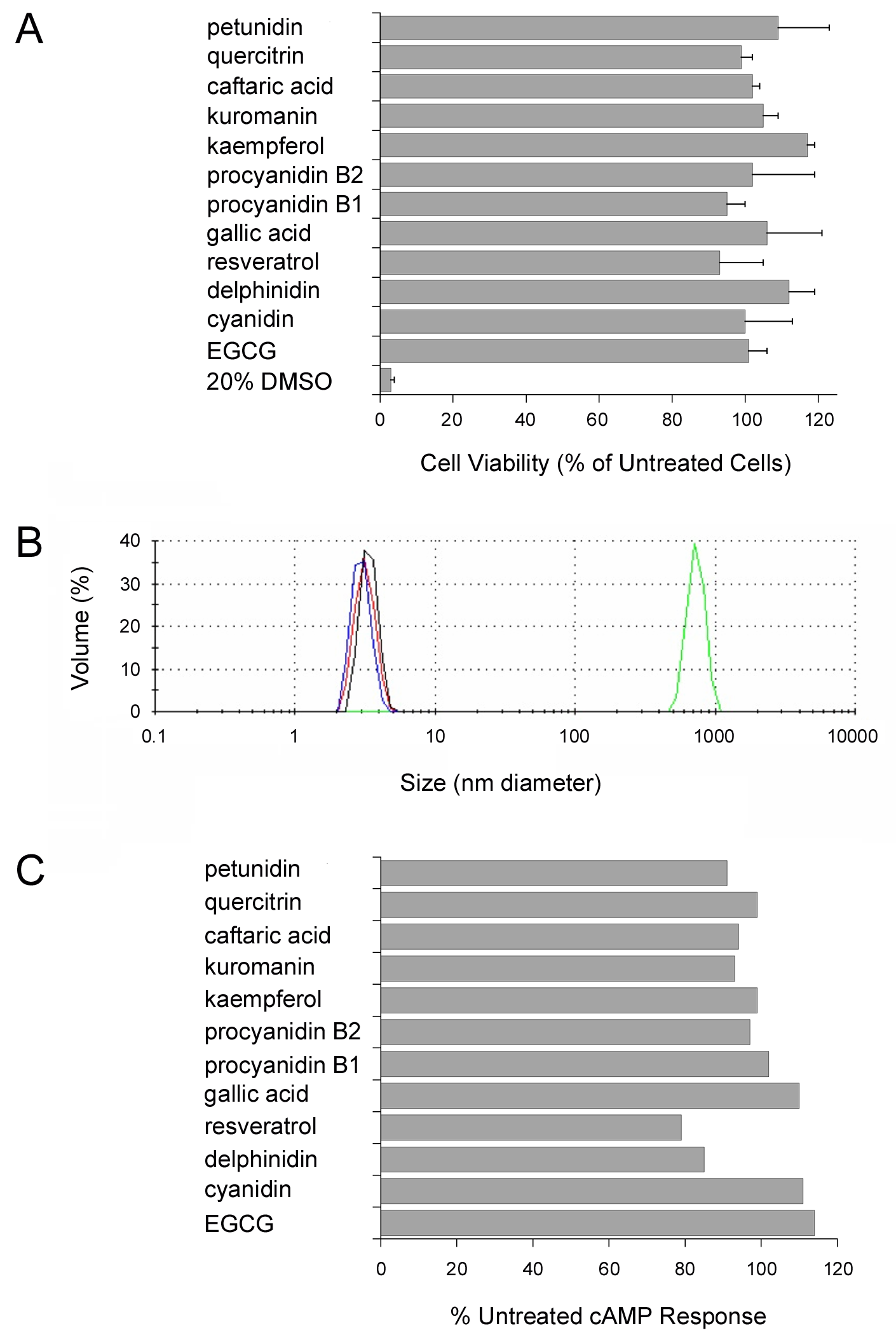

Supplement: S2 Fig — (A) CHO cells were incubated for 18 h with 10 μg/mL of the indicated compound or 20% DMSO before cell viability was determined with an MTS assay. Results were expressed as percentages of the MTS signal from untreated CHO cells. Data represent the avgs. ± std. devs. of 3 experiments or avgs. ± ranges of 2 experiments for kaempferol, procyanidin B2, delphinidin, EGCG, and DMSO. (B) The hydrodynamic diameters of CT (red), CT mixed with 10 μg/mL EGCG (blue) or procyanidin B2 (black), or boiled CT (green) were assessed by dynamic light scattering. As shown for EGCG and procyanidin B2, none of the tested compounds altered the hydrodynamic size of CT. (C) CHO cells were incubated with forskolin and 10 μg/mL of the indicated compound for 2 h before detecting the adenylate cyclase-driven production of cAMP. (TIF) [file pone.0166477.s002.tif]

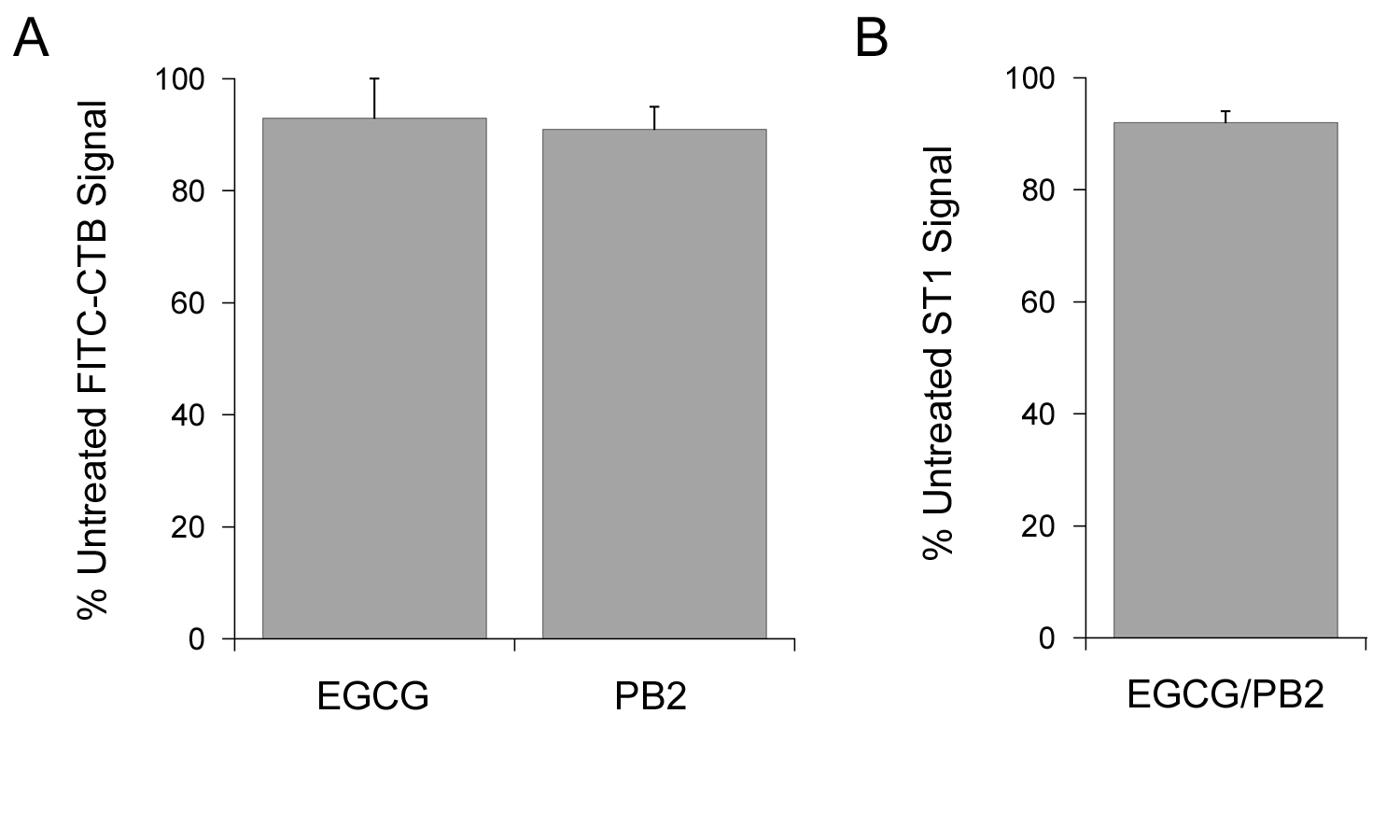

Supplement: S3 Fig — (A) Vero cells were incubated at 4°C for 30 min with 10 μg/mL of EGCG or PB2. The polyphenol was then removed from the medium and, after several washes, replaced with 1 μg/mL of FITC-CTB. After an additional 30 min at 4°C, unbound toxin was removed and FITC-CTB fluorescence was recorded with a plate reader. Values were standardized to the FITC-CTB signal from control cells that were not incubated with EGCG or PB2. (B) Vero cells were incubated for 1 h at 4°C with 0.5 μg/mL of ST1 and a cocktail containing 10 μg/mL each of EGCG and PB2. After subsequent incubations with anti-ST primary and AlexaFluor 488-conjugated secondary antibodies, the extent of ST1 binding was determined by fluorescent measurement with a plate reader. Values were standardized to the fluorescent signal from control cells that were exposed to ST1 in the absence of EGCG and PB2. Data from both panels represent the means ± SEMs of 3–4 independent experiments with 6 replicate wells per condition. (TIF) [file pone.0166477.s003.tif]

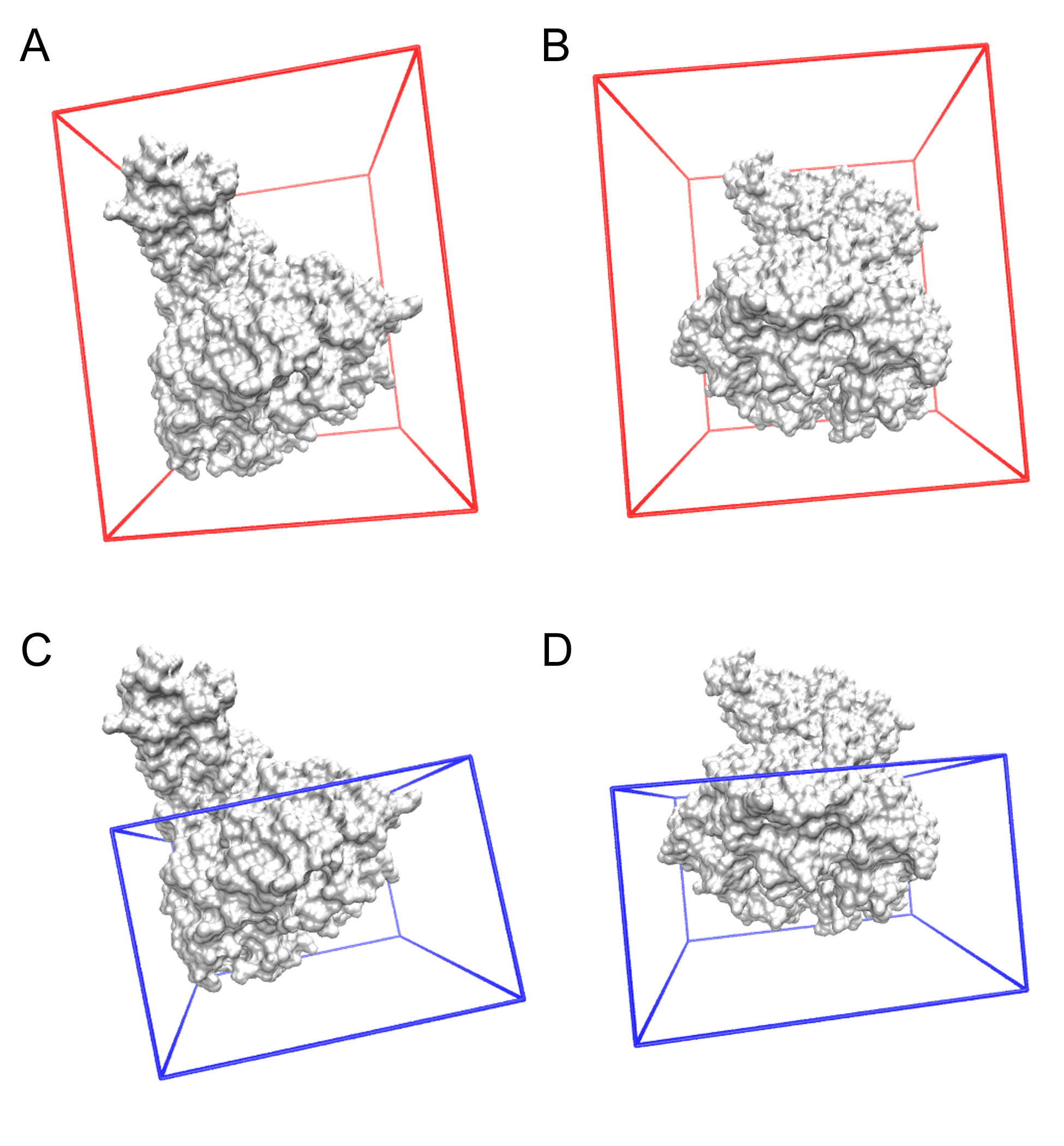

Supplement: S4 Fig — (A-B) The CT docking search box was defined by an unbiased large box (red) with center coordinates and sizes of (24, 0, 20.8) and (82, 74, 68), respectively. Panel B is rotated 90 degrees in relation to panel A. (C-D) A second round of docking used a more focused search box (blue) defined with center of (2.0, 0, 22.8) and size of (46, 74, 68). Panel D is rotated 90 degrees relative to panel C. (TIF) [file pone.0166477.s004.tif]

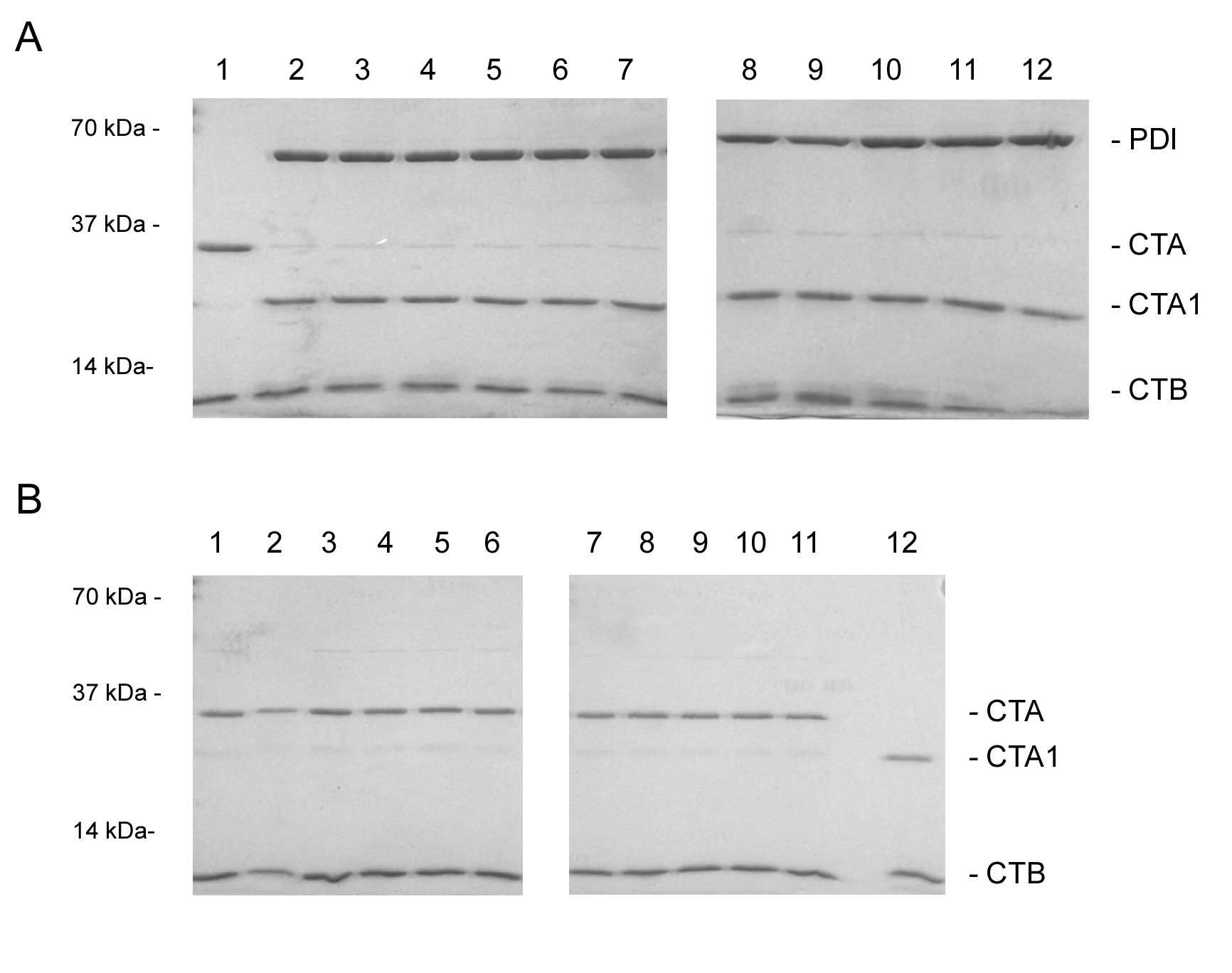

Supplement: S5 Fig — (A) CT was incubated with protein disulfide isomerase (PDI) for 1 h at 25°C in the presence of individual phenolic compounds before non-reducing SDS-PAGE with Coomassie staining was used to assess the redox status of the CTA subunit. Reduction of the CTA disulfide bond generates a 21 kDa CTA1 subunit and a 5 kDa CTA2 subunit; the CTB monomer is 11.5 kDa. Lane 1, CT alone; lanes 2–12, CT + PDI without added polyphenol (lane 2) or with 10 μg/mL PB2 (lane 3), kuromanin (lane 4), kaempferol (lane 5), gallic acid (lane 6), resveratrol (lane 7), quercitrin (lane 8), delphinidin (lane 9), cyanidin (lane 10), EGCG (lane 11), or PB1 (lane 12). (B) CT was incubated in the presence of individual phenolic compounds (10 μg/mL) for 1 h at 25°C before non-reducing SDS-PAGE with Coomassie staining was used to assess the redox status of the CTA subunit. Lane 1, untreated CT; lanes 2–12 CT treated with PB2 (lane 2), kuromanin (lane 3), kaempferol (lane 4), gallic acid (lane 5), resveratrol (lane 6), quercitrin (lane 7), delphinidin (lane 8), cyanidin (lane 9), EGCG (lane 10), PB1 (lane 11), or, as a positive control, β-mercaptoethanol (lane 12). (TIF) [file pone.0166477.s005.tif]

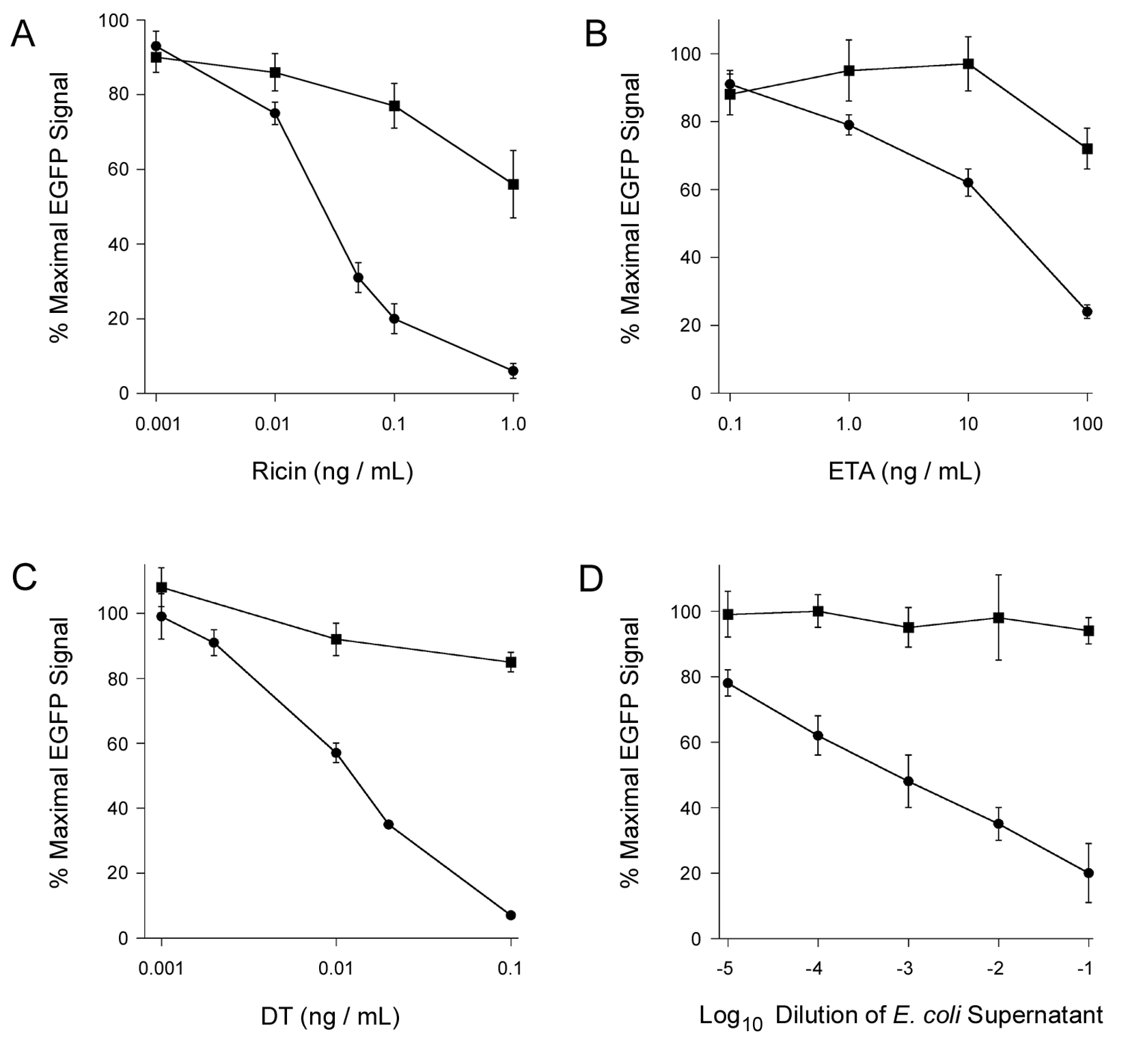

Supplement: S6 Fig — Vero-d2EGFP cells were co-incubated for 18 h in the absence (circles) or presence (squares) of 100 μg/mL of grape seed extract and various concentrations of (A) ricin, (B) ETA, (C) DT, or (D) ST1 and ST2 present in the cell-free culture supernatant of E. coli strain RM1697. For each experiment, results from six replicate wells per condition were expressed as percentages of the maximal EGFP signal recorded for unintoxicated Vero-d2EGFP cells. Data represent the means ± SEMs of at least 4 independent experiments for each toxin. (TIF) [file pone.0166477.s006.tif]

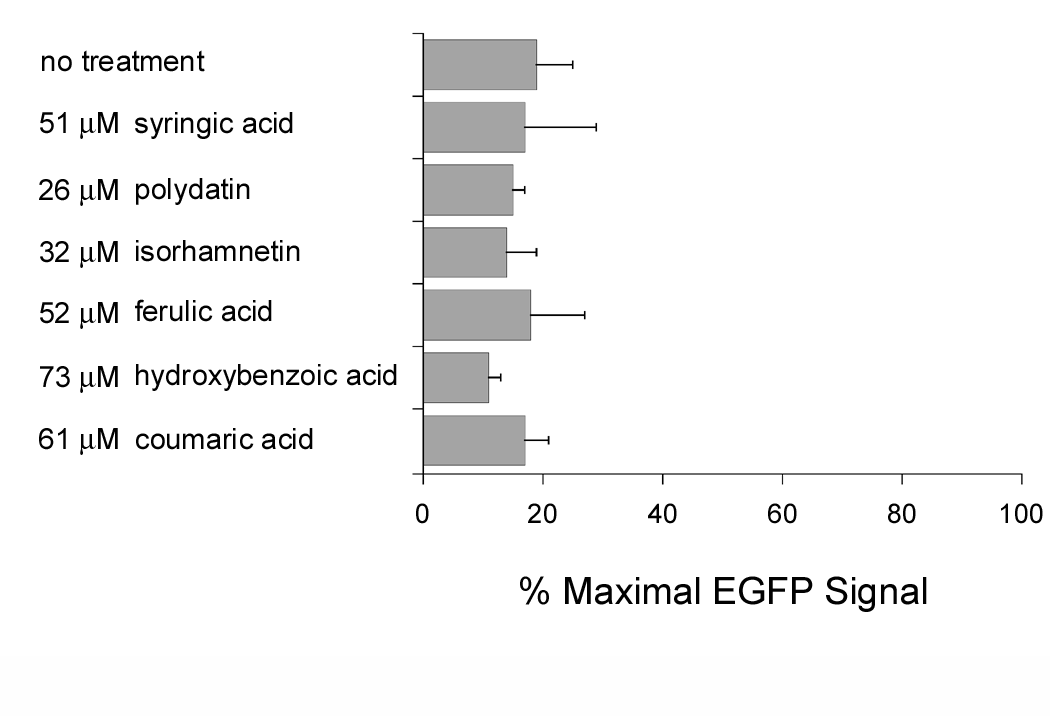

Supplement: S7 Fig — Vero-d2EGFP cells incubated with the listed concentrations of phenolic compound were challenged overnight with a ST1/ST2-containing cell-free culture supernatant from E. coli strain RM1697. The fluorescent signal from toxin-challenged cells was expressed as a percentage of the control EGFP signal recorded for unintoxicated cells incubated with the relevant phenolic compound. "No treatment" refers to toxin-challenged cells incubated in the absence of phenolic compound. Data represent the avgs. ± std. devs. of 3 independent experiments with 6 replicate samples. (TIF) [file pone.0166477.s007.tif]

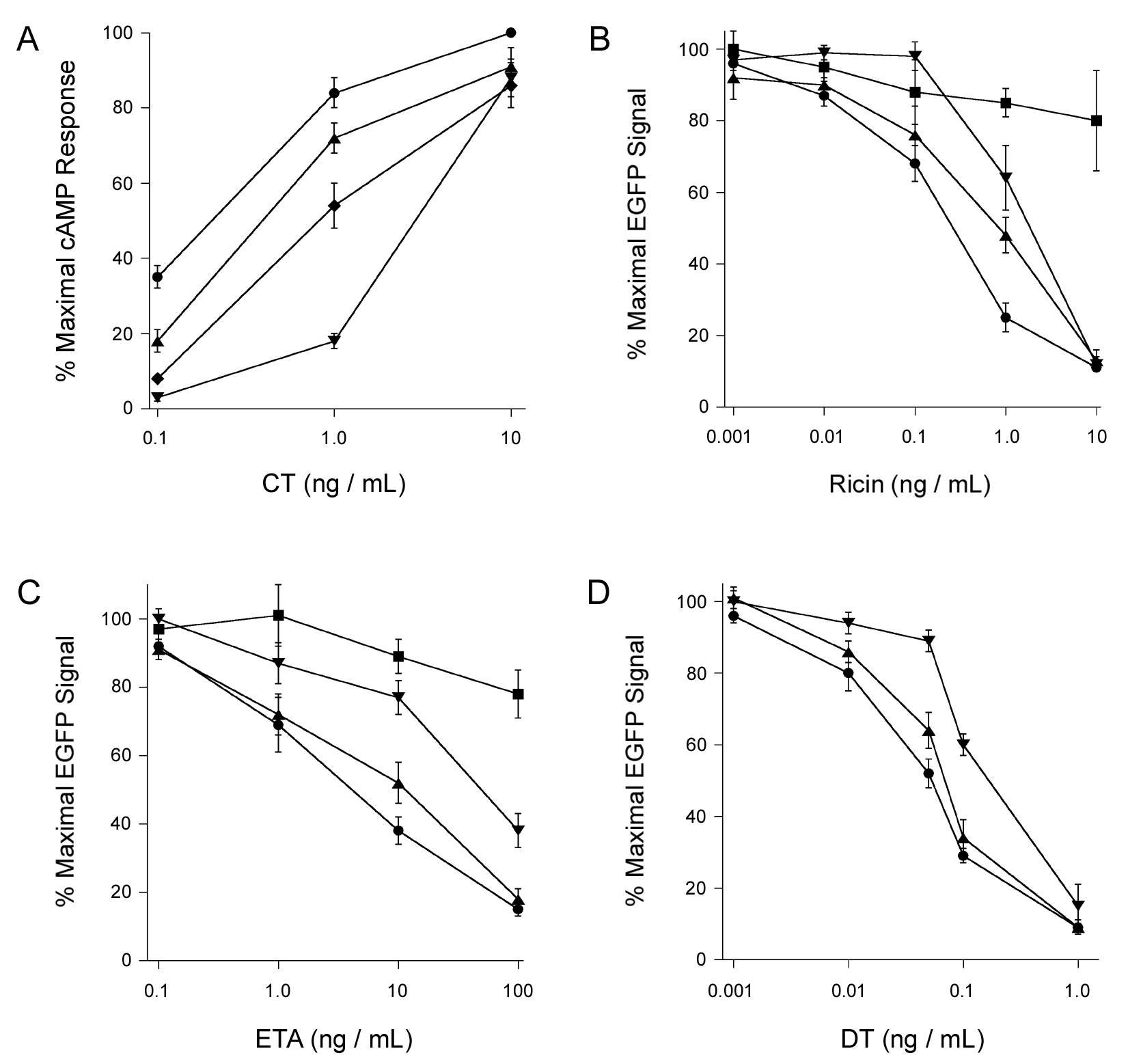

Supplement: S8 Fig — (A) CHO cells were exposed to varying concentrations of CT for 18 h in the absence or presence of EGCG before intracellular cAMP levels were quantified. Results from 3 replicate wells per condition were expressed as percentages of the maximal cAMP response from CHO cells incubated with 10 ng/mL of CT in the absence of EGCG. (B-D) Vero-d2EGFP cells incubated in the absence or presence of EGCG were challenged for 18 h with various concentrations of (B) ricin, (C) ETA, or (D) DT. Results from six replicate wells per condition were expressed as percentages of the maximal EGFP signal recorded for unintoxicated Vero-d2EGFP cells incubated with the corresponding concentration of EGCG. Circles, no EGCG present; squares, 4 μg/mL (8.8 μM) EGCG; inverted triangles, 1 μg/mL (2.2 μM) EGCG; diamonds, 0.5 μg/mL (1.1 μM) EGCG; triangles, 0.1 μg/mL (0.2 μM) EGCG. Data represent the means ± SEMs of at least 3 independent experiments for each toxin. (TIF) [file pone.0166477.s008.tif]
